# US Public Interest in Merkel Cell Carcinoma Following Jimmy Buffett’s Death and Implications for Continued Health Advocacy: Infodemiology Study of Google Trends

**DOI:** 10.2196/60282

**Published:** 2024-10-31

**Authors:** Macy Haight, Hayden R Jacobs, Sarah K Boltey, Kelly A Murray, Micah Hartwell

**Affiliations:** 1 Office of Medical Student Research, Oklahoma State University College of Osteopathic Medicine at the Cherokee Nation, 19500 E Ross St, Tahlequah, OK, 74464, United States, 1 8067360441; 2 Department of Emergency Medicine, Oklahoma State University Center for Health Sciences, Tulsa, OK, United States; 3 Department of Psychiatry and Behavioral Sciences, Oklahoma State University Center for Health Sciences, Tulsa, OK, United States

**Keywords:** skin cancer, merkel cell carcinoma, infodemiology, cancer, carcinoma, cell carcinoma, sunlight, infodemiology study, Google Trends, temporal analysis, United States, USA, sun

## Abstract

Through Jimmy Buffett’s unfortunate battle with lymphoma originating from Merkel cell carcinoma and subsequent media coverage of his death, public interest in skin cancer, Merkel cell carcinoma, and the health effects of sunlight exposure increased, as evidenced by our results.

## Introduction

Analyzing public interest in health-related topics through web-based search inquiries has become increasingly popular within the health care community over the past decade [1]. Infodemiology—the study of the “distribution and determinants of information across electronic platforms”—provides valuable insights into health information–seeking behavior [2]. Infodemiology research often relies on data from sources such as Google Trends, which aggregates and anonymizes search data from Google’s search engine. Unlike traditional data collection methods, Google Trends offers real-time data that can be stratified by geographical region and time period. Google Trends’ utility in quantifying public interest in health topics and identifying health information–seeking behavior trends has been studied, proving to be an effective means of evaluating public interest in health-related topics [3].

With the rise of social media and celebrities’ transparency regarding personal health issues, infodemiology provides a means of quantifying public interest in health information across electronic platforms [4]. Celebrity health events have been shown to significantly impact public interest in specific diseases and health behaviors. The “Angelina Jolie effect,” for example, led to a surge in referrals to breast cancer clinics and genetics services following the actress’ decision to undergo a preventive double mastectomy. Similarly, media coverage of celebrity cancer diagnoses and deaths has been linked to increased public interest in cancer-related topics [5,6].

Singer-songwriter Jimmy Buffett, who famously sang “Some of it’s magic, some of it’s tragic, but I had a good life all the way,” tragically died on September 1, 2023, after battling lymphoma precipitating from Merkel cell carcinoma (MCC) [7,8]. Buffett’s death received widespread media coverage, prompting renewed interest in this rare disease among the general public [9]. To investigate the impact of celebrity health events on public interest regarding specific health-related topics, we conducted an analysis of search interest surrounding this rare and aggressive skin cancer, using Google Trends.

## Methods

### Study Design

Google Trends was used to quantify search interest in “skin cancer,” “Merkel cell carcinoma,” and “health effects of sunlight exposure” in all US regions for a 60-day period encompassing Buffett’s death. Daily relative search interest (RSI) data were extracted from Google Trends from August 2 through September 30, 2023. RSI is a value from 0 to 100 based on the highest volume criteria within the search. An autoregressive integrated moving algorithm was trained on data from August 2 through September 2, 2023, to forecast daily search volume and 95% CIs for September 3 through September 30, 2023, as if the event did not occur. We then compared forecasted values to the actual values to note peak changes during the time frame and where the actual RSI falls outside the forecasted 95% CI to identify statistically significant values (*P*<.05).

### Ethical Considerations

This study did not involve human subjects, as defined under US Department of Health and Human Services regulations 45 CFR Part 46. Therefore, institutional review board approval was not required. We used publicly available, deidentified data from Google Trends, which aggregates search behavior from large populations and ensures that all information is anonymized, with no personal identifiers or sensitive data associated with individual users.

## Results

**Figure 1.**
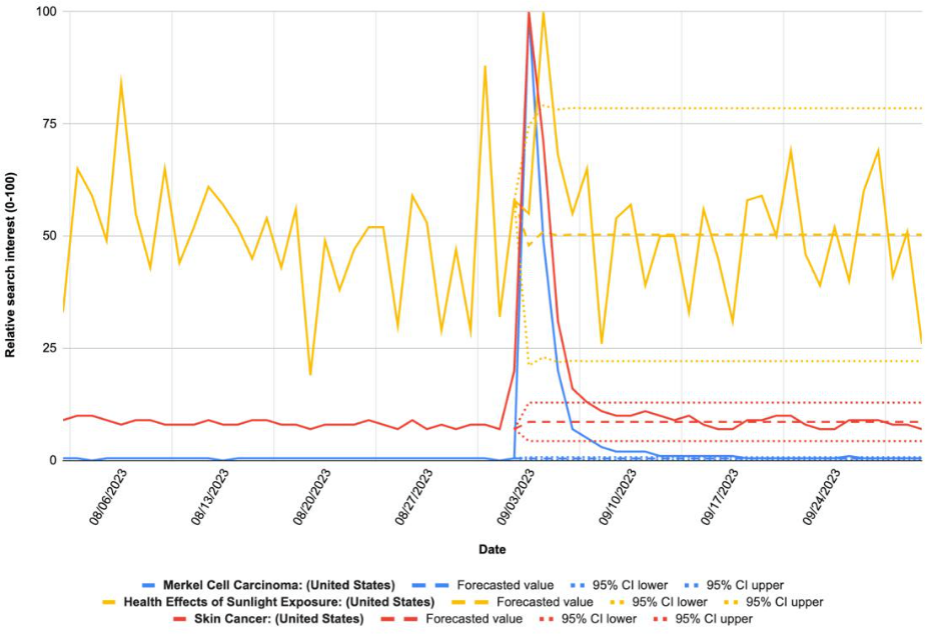
Relative search interest for “skin cancer,” “Merkel cell carcinoma,” and “health effects of sunlight exposure” in the United States by date.

Our analysis revealed statistically significant increases in search interest for MCC and related topics following Buffett’s death (Figure 1 and Table 1). Peak search interest occurred 3 days after Buffett’s death (RSI=100), with sustained interest observed up to 15 days after the event. Percent differences between the actual and projected RSI values for the search terms during this 15-day period ranged from 95.79% to 21,968.97%, indicating the substantial impact of Buffett’s death on public awareness of MCC.

**Table 1.** Relative search interest (RSI) percent difference by search term, with 95% CIs for a 60-day period encompassing Buffett’s death.

	Forecasted RSI (95% CI)	Actual RSI	% difference
“Skin Cancer”	8.63 (4.33‐12.92)	100	1059.42
“Merkel Cell Carcinoma”	0.45 (0.17‐0.74)	100	21,968.97
“Health Effects of Sunlight Exposure”	51.07 (23.04‐79.11)	100	95.79

## Discussion

Our analysis of temporal search interest trends provides insights into the immediate impact of a celebrity’s health event on public engagement with skin cancer information. The sustained interest in MCC following Buffett’s death highlights the potential crucial role of infodemiology research in understanding impacts of celebrity health events on public health behaviors. By leveraging tools such as Google Trends, researchers can gain valuable insights into health information–seeking behavior patterns and identify opportunities for targeted public health interventions.

Although celebrity endorsements and media coverage can raise short-term awareness, sustained efforts are needed to ensure that awareness translates into meaningful action. By monitoring health information–seeking behavior trends, researchers can identify areas where targeted interventions and strategies are needed to promote long-term behavioral change. As we navigate the complex interplay between media influence and public health, our study contributes to ongoing discussions on optimizing strategies for increasing awareness and improving health outcomes associated with diseases, such as MCC.

## Abbreviations

MCC: Merkel cell carcinoma
RSI: relative search interest

## References

[1] Mavragani A, Ochoa G (2019). Google Trends in infodemiology and infoveillance: methodology framework. JMIR Public Health Surveill.

[2] Eysenbach G (2009). Infodemiology and infoveillance: framework for an emerging set of public health informatics methods to analyze search, communication and publication behavior on the internet. J Med Internet Res.

[3] Cohen SA, Cohen LE, Tijerina JD (2020). The impact of monthly campaigns and other high-profile media coverage on public interest in 13 malignancies: a Google Trends analysis. Ecancermedicalscience.

[4] Mavragani A (2020). Infodemiology and infoveillance: scoping review. J Med Internet Res.

[5] Kaleem T, Malouff TD, Stross WC (2019). Google search trends in oncology and the impact of celebrity cancer awareness. Cureus.

[6] Evans DG, Barwell J, Eccles DM (2014). The Angelina Jolie effect: how high celebrity profile can have a major impact on provision of cancer related services. Breast Cancer Res.

[7] (2023). Jimmy Buffett (1946-2023). Jimmy Buffett.

[8] Lyrics. Jimmy Buffett.

[9] Kopec V Jimmy Buffett’s death puts merkel cell carcinoma in the spotlight. The Skin Cancer Foundation.

